# Efficient Breeding of Early-Maturing Rice Cultivar by Editing *PHYC* via CRISPR/Cas9

**DOI:** 10.1186/s12284-021-00527-3

**Published:** 2021-10-13

**Authors:** Bin Li, Xi Du, Yunyan Fei, Fangquan Wang, Yang Xu, Xia LI, Wenqi Li, Zhihui Chen, Fangjun Fan, Jun Wang, Yajun Tao, Yanjie Jiang, Qian-Hao Zhu, Jie Yang

**Affiliations:** 1 Institute of Life Science, Jiangsu University, Zhenjiang, 212013 Jiangsu China; 2 Institute of Food Crops, Jiangsu Academy of Agricultural Sciences/Nanjing Branch of Chinese National Center for Rice Improvement, Nanjing, 210014 Jiangsu China; 3 Jiangsu Co-Innovation Center for Modern Production Technology of Grain Crops, Yangzhou University, Yangzhou, 225009 Jiangsu China; 4 CSIRO Agriculture and Food, GPO Box 1700, Canberra, ACT Australia

Rice (*Oryza sativa* L.) is a facultative short-day plant and provides staple food for more than half of the human population. Heading date (also known as flowering time) is one of the most important agronomic traits of rice as it determines the regional and seasonal adaptability of rice varieties and has a significant influence on the grain yield (Zhou et al. 2021). To maximize rice production, it is crucial to breed rice cultivars with optimum heading date suitable for the cropping areas where the cultivars are to be used. Domestication and breeding activities had created diverse natural variations of flowering time and artificially modulated the flowering regulatory pathways of rice, which helped significant expansion of rice cultivating areas (Goretti et al. 2017; Itoh et al. 2018; Hu et al. 2019). The genetic pathways regulating rice flowering time have been extensively investigated and a number of flowering time genes have been cloned and used to modify rice heading date using traditional and state-of-art molecular methods, such as cross-based introgression of key heading date gene(s) with the assistance of molecular markers and molecular modulation of the expression levels of heading date genes by gene editing (Zhou et al. 2021).

Rice has a complex genetic network regulating flowering time (Hori et al. 2016; Zhou et al. 2021). One of the major components of the network is phytochromes, including *PHYA*, *PHYB*, and *PHYC*. They are regulators of *Ghd7*, a key floral repressor in rice. *PHYA* alone or a combination of *PHYB* and *PHYC* can induce the expression level of *Ghd7* (Osugi et al. 2011). The function of PHYC depends on the PHYB protein which participates in the regulation of PHYC expression level and in photomorphogenesis via PHYB/PHYC heterodimer (Osugi et al. 2011; Xie et al. 2014). While, under long-day (LD) conditions, *phyA* single mutation hardly affects flowering time of rice, *phyB* or *phyC* single mutant flowers ~ 12 days earlier than the wild-type (Takano et al. 2005, 2009).

Nanjing46 (NJ46 for short), ranked as one of the top varieties for its palatability by consumers in the triangle region of the Yangtze River, is a low amylose content rice variety suitable for planting in southern Jiangsu, China. Northward expansion of NJ46 requires shortening its long growth duration by promoting early flowering without yield penalty. In a previous study, we generated early-maturing japonica rice lines (on the Nanjing9108 genetic background) by CRISPR/Cas9-mediated editing of *Hd2*, *Hd4*, and *Hd5* genes, but the lines flowered too early to be suitable for planting in Jiangsu (Li et al. 2017). We were thus seeking alternative genes for fine tuning the heading date and considered *PHYC* as a suitable candidate based on its moderate function in regulating flowering time (Takano et al. 2005; Osugi et al. 2011).

In this study, we used the CRISPR/Cas9 gene editing system to knock out *PHYC* in NJ46. To generate loss-of-function *PHYC* mutants, we designed guide RNA targeting the first exon of *PHYC* (Fig. 1A). We generated 20 independent T_0_ plants using *Agrobacterium*-mediated transformation approach. Positive T_0_ transgenic plants were identified by PCR amplification of a fragment of the *Hyg* gene that was used as a selection marker. The target genomic region of *PHYC* was amplified by a pair of primers (*PHYC*-F/R; Additional file 1: Table S1) flanking the target site and sequenced. The sequencing results were decoded by the Degenerate Sequence Decoding (DSD) method (Liu et al. 2015) (Fig. 1A, Additional file 1: Fig. S1A). Based on screening of the absence of *Hyg* and *Cas9* and the presence of mutation in the target site using PCR in T_1_ generation, we found two transgene-free homozygous gene editing lines (both with a single base insertion that caused frame shift and pre-mature stop codon; Additional file 1: Fig. S1B) and named them *phyC-1* and *phyC-2*. We analyzed the phenotypes of homozygous T_1_ and T_2_ populations, together with the wild-type NJ46, by recording heading date and other agronomic traits and evaluating rice grain quality under natural conditions (same as LD conditions).

**Fig. 1:**
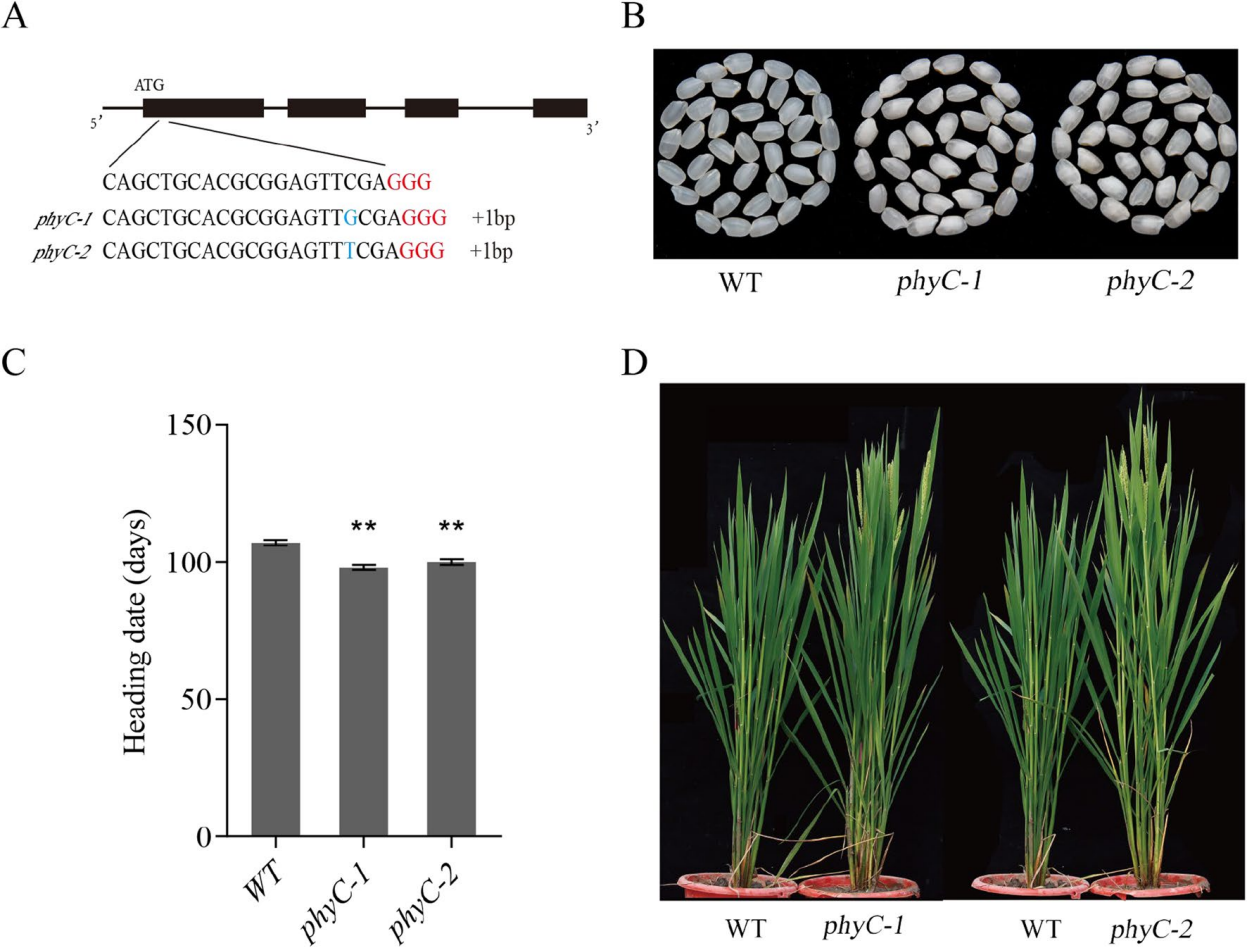
Characterization of early-maturing rice generated by CRISPR/Cas9-mediated editing of *PHYC*. **A** Schematic diagram of the *PHYC* gene and the position of the editing target site. Black boxes and lines in between represent exons and introns, respectively. The target sequence is shown in black and the PAM sequence (CGG) in red. Underneath the target sequence is the sequence alignment to show the 1-bp insertion (highlighted in blue) induced by gene editing in the two independent transgenic lines (*phyC-1* and *phyC-2*). **B** Comparison of the morphology of the milled rice grains from T_2_ plants. **C** Comparison of heading date between the two mutants and the wild-type. **D** Plant morphology of the *PHYC* mutants (*phyC-1* and *phyC-2*; T_2_ generation) and NJ46 at the flowering stage

The heading date of *phyC-1* and *phyC-2* was about 7 days earlier than the wild-type (Fig. 1C, D). It has been reported that in addition to the regulation of heading date, *PHYC* is also involved in the regulation of chlorophyll content and leaf angle in rice seedlings, plant height, panicle architecture, and grain size (Li et al. 2019). We thus compared these traits between the mutants and wild-type in 2019 and 2020. In both T_1_ and T_2_ generations, no significant difference in plant height and panicle length was observed between the mutants and the wild-type (Additional file 1: Fig. S2). Compared to the wild-type, the mutants showed a slight but significant increase of grain length and 1000-grain weight, but no difference in grain width and grain thickness (Additional file 1: Fig. S3). The obviously noticeable difference was the appearance quality of polished rice grains after shelling (Fig. 1B). The possible reason for this phenomenon was that the transparency of mutants of the semi-glutinous variety NJ46 was decreased due to the higher level of chalkiness degree and the low water content (Additional file 1: Fig. S4A, B). Gel consistency and amylose content are two important traits for evaluating the eating quality of rice. There was no difference in gel consistency between the mutants and the wild-type (Additional file 1: Fig. S4C). However, the amylose content of *phyC-1* and *phyC-2* decreased by 13.6% and 12.7%, compared with that of the wild-type (Additional file 1: Fig. S4D). Decrease of appearance quality and amylose content in *phyC-1* and *phyC-2* might be a result of cooccurrences of high temperature in the early stage of grain-filling due to the advanced heading stage (Nevame et al. 2018; Hirano and Sano 1998; Ahmed, et al. 2015) (Additional file 1: Fig. S5).

These results indicated that knockout *PHYC* by CRISPR/Cas9-mediated gene editing could moderately shorten the heading date of NJ46, making it suitable for cultivation in northern Jiangsu, although probably would have some negative influences on grain quality. But the slightly adverse effects of loss-of-function of *PHYC* on the appearance quality of rice caryopses may be eliminated if the novel germplasm is planted in the regional with more suitable light and temperature conditions to avoid high temperatures during grain filling stage. We will address this issue in the follow-up studies.

In this study, we demonstrated that the CRISPR/Cas9-mediated gene editing approach is an effective tool for manipulating heading date in rice, consistent with the results by Cui et al. (2019), who investigated the usage of gene editing in alteration of flowering time by targeting 10 heading time genes. Given that rice yield is positively correlated with growth duration, or days to heading, it is important to balance the trade-off between yield and early flowering. We envision that the balance can be achieved by choosing suitable genes and appropriate approaches for manipulating their functions. While a number of genes with a known function in regulating rice flowering time have been or are being investigated using the gene editing technology, more genes, single or in different combinations, with micro-effects on heading date changes, should be investigated in future studies in order to breed novel elite varieties suitable for different cultivation areas. Base editing and prime editing can be used to precisely alter gene sequence (Komor et al. 2016; Anzalone et al. 2019) and have been demonstrated to be promising in crop improvement, such as fine-tuning of amylose content (Xu et al. 2021; Li et al. 2020). Application of precise editing technology in the right genes will finally help us achieve the goal of fine-tuning the heading date of rice without penalty in yield and quality.

## Supplementary Information

**Additional file 1.** Materials, methods and data.

## Data Availability

All data generated or analyzed during this study are included in this published article and its supplementary information files.

## Abbreviations

*PHYA*: Phytochrome A
*PHYB*: Phytochrome B
*PHYC*: Phytochrome C
*Ghd7*: Grain number, plant height and heading date 7
LD: Long-day
*Hd2*: Heading date 2
*Hd4*: Heading date 4
*Hd5*: Heading date 5
WT: Wild type
CRISPR: Clustered regulatory interspaced short palindromic repeat
sgRNA: Single guide RNA
CTAB: Cetyltrimethylammonium Bromide
*Hyg*: Hygromycin
