# Supplementary material for: Efficient Breeding of Early-Maturing Rice Cultivar by Editing PHYC via CRISPR/Cas9
Source: Rice (N Y). 2021 Oct 13;14:86. doi: 10.1186/s12284-021-00527-3 (PMC8514591; doi:10.1186/s12284-021-00527-3)
Supplement: Supplementary file 1 — Additional file 1. Materials, methods and data. [file 12284_2021_527_MOESM1_ESM.doc]

**Supplementary data**

**Materials and methods**

**Plant materials and growth conditions**

Wild-type NJ46, a japonica rice variety (*Oryza sativa* L. ssp*. japonica*), was used as the target material for CRISPR/Cas9-mediated gene editing of the *PHYC* gene and grown in parallel with transgenics for phenotyping. Seeds were sown in seed beds in a greenhouse in early April and transplanted to the paddy field of Jiangsu Academy of Agricultural Sciences in the middle of May. The planting density was 15 cm between plants and 20 cm between rows. Rice was cultivated in the paddy field following the normal agricultural practices.

**Vector construction and transformation**

Under the guidance of CRISPR Primer Designer (http://www.plantsignal.cn), the target sequence of gene editing was selected and the target sequence-containing chimeric primers were designed (Yan et al., 2015). The CRISPR/Cas9 expression vector was constructed according to the protocol previously described (Ma et al., 2015). Briefly, the target sequence flanked by joint sequences was biosynthesized and inserted in the *Bsa* I site of the sgRNA expression cassette. The sgRNA expression cassette integrated with the sgRNA targeting *PHYC* was then amplified by nested PCR. After digestion with *Bsa* I, the sgRNA expression cassette was assembled into the linearized pYLCRISPR/Cas9-MH vector in the *Bsa* I site using T4 DNA ligase. The vector was transformed into *E. coli* strain DH5α and confirmed by sequencing.

**Mutation detection in T_0_ transgenic plants**

The constructed final vector was introduced into NJ46 using Agrobacterium strain EHA105 by *Agrobacterium*-mediated transformation (Hiei et al., 1997). To identify positive transformants (T_0_ generation), genomic DNA was extracted from leaves using the CTAB method (Murray and Thompson, 1980). The positive transgenic plants were identified by PCR amplification of a *Hyg* fragment using the primers *Hyg*-F and *Hyg*-R. *Hyg*-F/R and *Cas9*-F/R primers were used to screen transgene-free mutants in T_1_ generation. To detect mutations in the target site, a pair of gene specific primer *PHYC*-F/R was used to amplify the target genomic regions. The obtained PCR products were sequenced and decoded by the DSDecode program to identify mutations (http://skl.scau.edu. cn/dsdecode) (Liu et al., 2015). At T_1_ generation, based on the mutation type identified in the T_0_ generation, homozygous editing plants from two independent transformants were chosen for further analysis. Homozygous T_2_ lines were grown in rows to investigate the heading date and other agronomic traits. Sequences of all the primers used in this study are listed in Supplemental Table 1.

**Measurement of rice agronomic traits**

Heading date, the number of days from seed sowing to the appearance of the first panicle, of T_1_ and T_2_ plants was recorded. Other agronomic traits of T_1_ and T_2_ plants were measured at the maturity stage. The plant height was measured with a ruler in the field. Measurement of the grain size was done with a vernier caliper (Mitutoyo, Japan). Chalkiness degree and chalky grain rate were measured by rice quality analyzer (Wanshen corporation, China). The content of amylose was determined by iodine blue colorimetry.

**References**

Murray M.G, Thompson W.F. (1980) Rapid isolation of high molecular weight plant DNA. Nucleic Acids Res 8(19): 4321–4326

Hiei Y, Komari, T. & Kubo T. (1997) Transformation of rice mediated by Agrobacterium tumefaciens. Plant Mol Biol 35: 205–218

Ma X, Zhang Q, Zhu Q, Liu W, Chen Y, Qiu R, Wang B, Yang Z, Li H, Lin Y, Xie Y, Shen R, Chen S, Wang Z, Chen Y, Guo J, Chen L, Zhao X, Dong Z, Liu Y-G. (2015) A robust CRISPR/Cas9 system for convenient, high-efficiency multiplex genome editing in monocot and dicot plants. Mol Plant 8: 1274–1284

Yan M, Zhou S.R, Xue H.W. (2015) CRISPR Primer Designer: Design primers for knockout and chromosome imaging CRISPR-Cas system. J. Integr. Plant Biol 57: 613-617


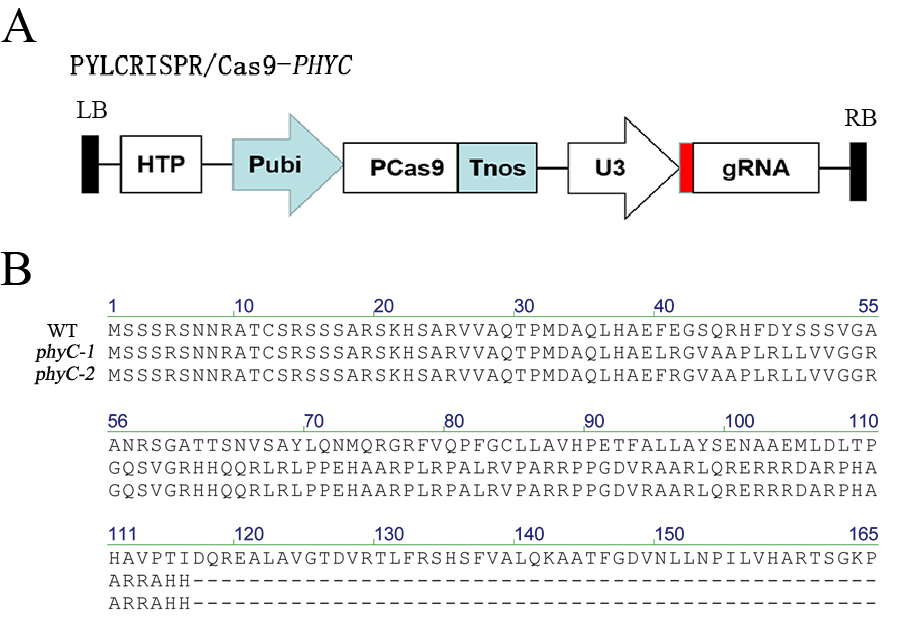


**Fig. S1. Vector structure and protein sequence prediction**

A, Structure of the PYLCRISPR/Cas9-*PHYC*. B, The predicted protein sequence. Dotted lines indicate termination of translation.


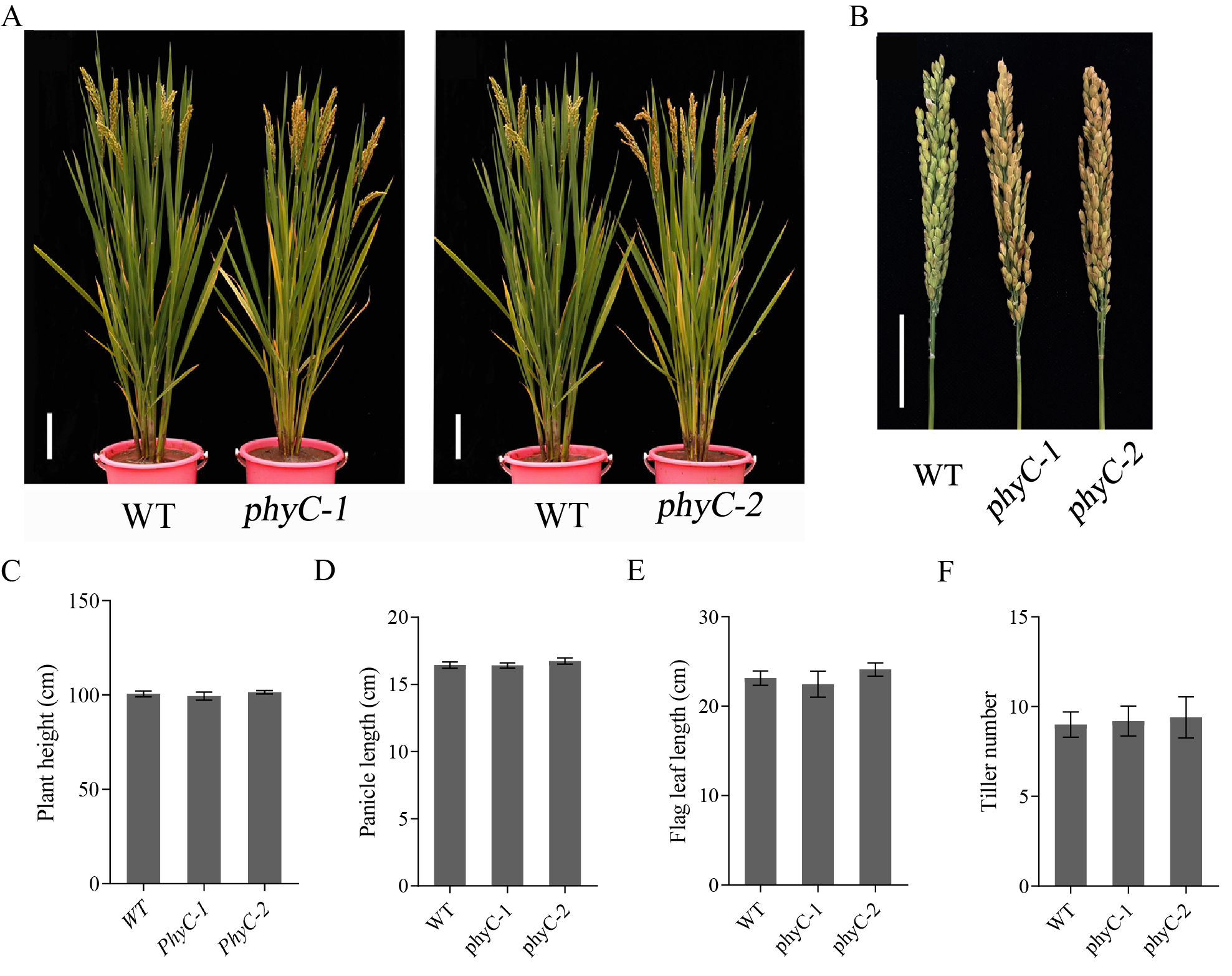


**Fig. S2. Other agronomic traits**

A and C, Plant height. Scale bars, 10cm. B and D, Panicle length. Scale bars, 5cm. E, Flag leaf length. F, Tiller number. Values are Mean ± SD (n = 10). *P* values were calculated by the Student’s t test (ns, no significant; *, *P* < 0.05; **, *P* < 0.01).


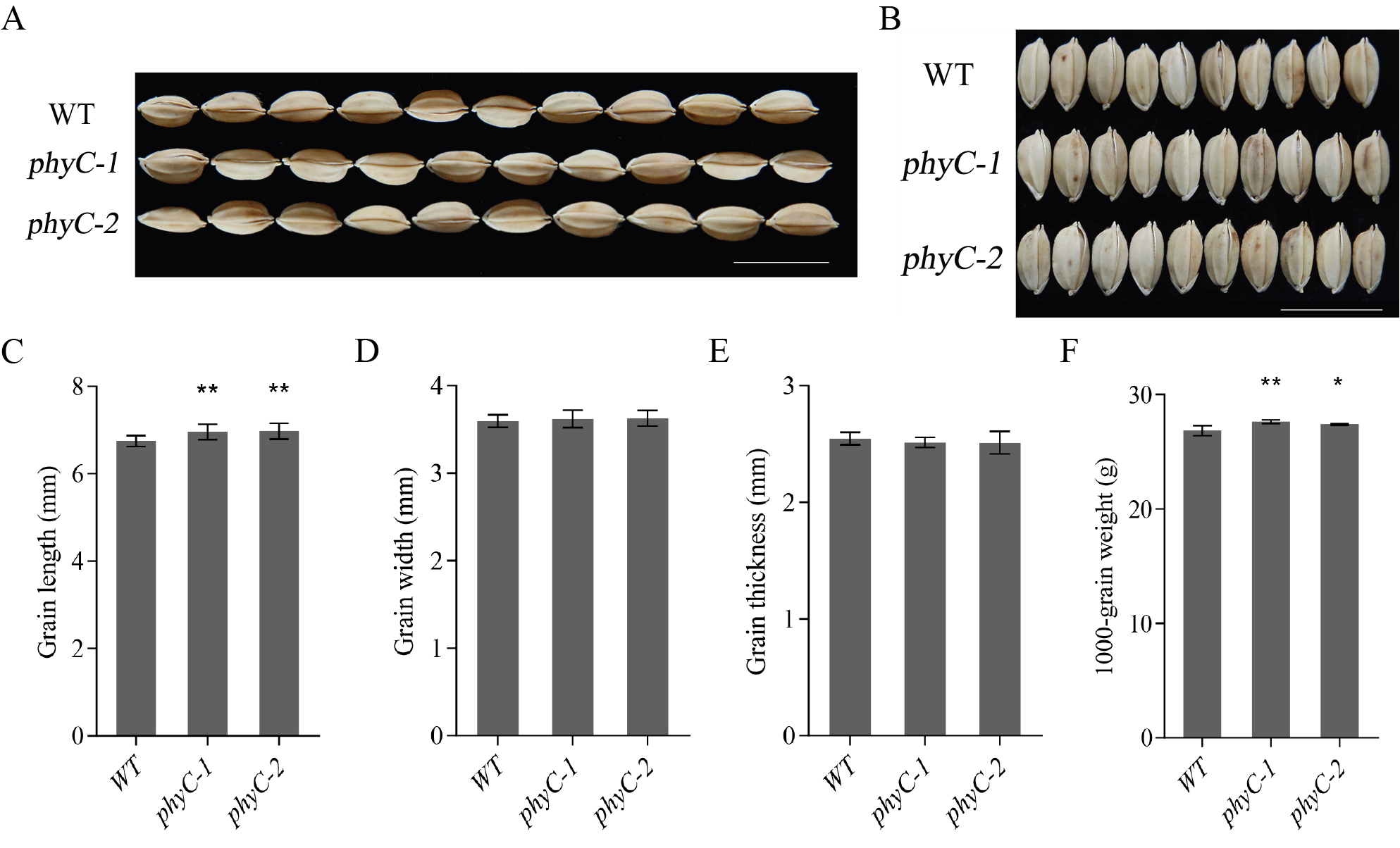


**Fig. S3. Grain size**

A and B, Mature grain phenotype. Scale bars, 10 mm. C, Grain length. D, Grain width. E, Grain thickness. F, 1000-grain weight. Values are Mean ± SD (n = 10).

*P* values were calculated by the Student’s t test (*, *P* < 0.05; **, *P* < 0.01).


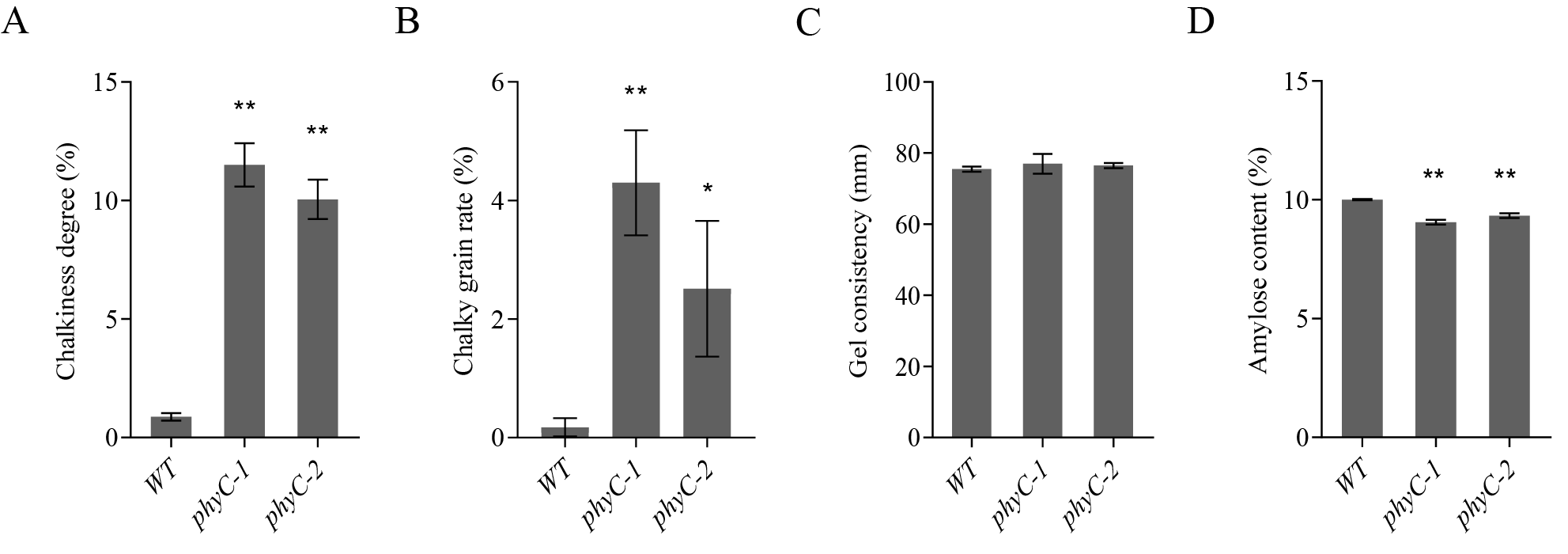


**Fig. S4. Rice grain quality characteristics**

A, Chalkiness degree. B, Chalky grain rate. C, Gel consistency. D, Amylose content. Values are Mean ± SD (n = 3). *P* values were calculated by the Student’s t test (*, *P* < 0.05; **, *P* < 0.01).


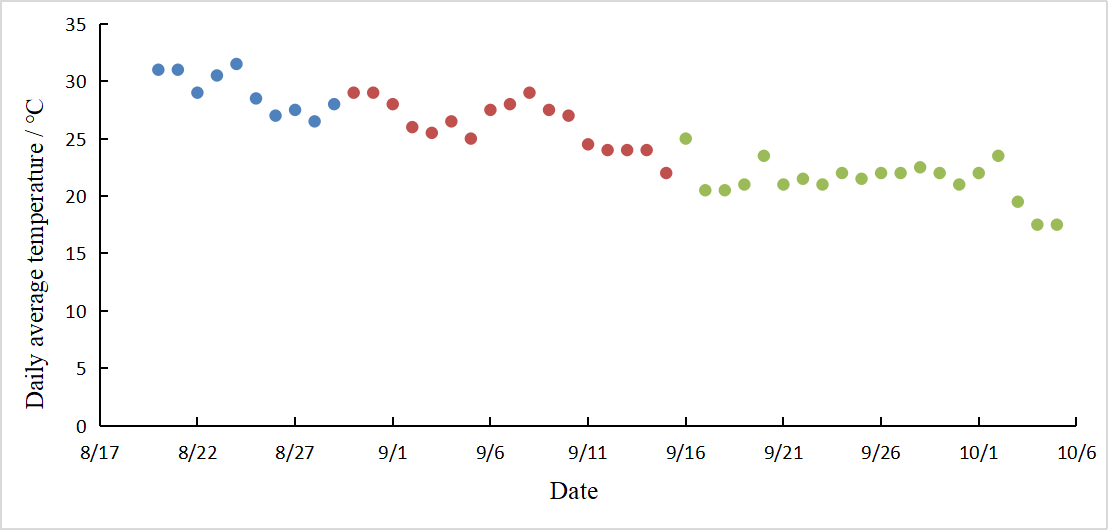


**Fig. S5. Daily average temperature during grain-filling period in 2020.**

Mutants and WT are indicated by green dots and red dots. Blue dots indicate overlap between WT and mutants. The initial green and blue dots represent the flowering time of mutants and WT.

| Primers name | Primer sequence(5’-3’) |
| --- | --- |
| U-F | CTCCGTTTTACCTGTGGAATCG |
| U-R | CGGAGGAAAATTCCATCCAC |
| *PHYC*-F | CCATGATTCCCCGCTGATACC |
| *PHYC*-R | GTACTCCTCAGACTGCCTGATCTG |
| *Hyg*-F | TCCGGAAGTGCTTGACATT |
| *Hyg*-R | GTCGTCCATCACAGTTTGC |
| *Cas9*-F | AGCGGCAAGACTATCCTCGACT |
| *Cas9*-R | TCAATCCTCTTCATGCGCTCCC |

**Supplemental Table 1. Oligonucleotides used in this study**
